# Impact of concomitant tricuspid annuloplasty on right ventricular remodeling in patients with rheumatic mitral valve disease

**DOI:** 10.1186/s12947-021-00245-2

**Published:** 2021-03-04

**Authors:** Yue Zhong, Wenjuan Bai, Hui Wang, Hong Qian, Li Rao

**Affiliations:** 1 Department of Cardiology, West China Hospital of Sichuan University, 37 Guo Xue Xiang, Chengdu, 610041 Sichuan China; 2 Department of Cardiovascular Surgery, West China Hospital of Sichuan University, Chengdu, 610041 Sichuan China

**Keywords:** Right ventricular remodeling, Functional tricuspid regurgitation, Rheumatic mitral valve disease, Concomitant tricuspid valve annuloplasty

## Abstract

**Background:**

Studies on the management of functional tricuspid regurgitation (TR) during mitral valve operations have drawn inconsistent conclusions. This study was designed to compare the treatment strategy of concomitant tricuspid annuloplasty (TAP) against isolated mitral valve replacement (MVR) in rheumatic mitral valve disease patients, and to assess the effect of concomitant TAP on postoperative right ventricular (RV) remodeling and function.

**Methods:**

One hundred-seventy patients with rheumatic mitral valve disease receiving MVR were categorized into TAP group (*n* = 124) and non-TAP group (*n* = 46). Clinical and echocardiographic data were collected preoperatively and at 1-year follow-up. Three-dimensional echocardiographic indices of RV geometry and function were analyzed.

**Results:**

At baseline, concomitant TAP group had larger RV end-diastolic volume, more decreased RV ejection fraction and RV longitudinal strain than non-TAP group (all *P* <  0.001). At 1-year follow-up, TAP group had improved RV geometry and function. While adverse changes were observed in non-TAP group. In analysis of variance, the above indices demonstrated significant interaction with different treatment group (all *P* <  0.001). In multivariate regression analysis, independent of age and Maze procedure, concomitant TAP was associated with postoperative RV volume reduction (*P* <  0.001), improvement of RV ejection fraction (*P* <  0.001), and relieved postoperative functional TR severity (*P* = 0.025).

**Conclusions:**

Our results suggest that concomitant TAP could improve RV remodeling and function for rheumatic mitral valve disease patients, while those with mild preoperative functional TR who had isolated MVR might experience RV dilation and deterioration of RV function at follow-up. Concomitant surgery for functional TR could be considered for patients undergoing MVR with rheumatic mitral valve disease.

**Supplementary Information:**

The online version contains supplementary material available at 10.1186/s12947-021-00245-2.

## Introduction

Concomitant tricuspid annuloplasty (TAP) at the time of mitral surgery has gained growing acceptance recently. Despite surgical repair for severe tricuspid regurgitation (TR) at the time of left-sided valve surgery is a Class I recommendation under the current guidelines, the optimal management for moderate and mild functional TR depends more on whether the patient had dilated tricuspid annulus and/or right heart failure [1, 2]. Besides, patients with rheumatic heart disease had higher rates of developing late postoperative TR, those patients with preoperative significant TR demonstrate a survival benefit from concomitant TAP [3]. Rheumatic valve disease patients undergoing mitral valve replacement (MVR) might need more aggressive treatment for functional TR, but the threshold value for intervention is ambiguous. Previous studies mostly focused on degenerative and functional mitral valve disease, which have shown that for moderate and severe preoperative TR, concomitant TAP could improve postoperative right ventricular (RV) function, leading to favorable RV remodeling [4, 5]. However, data regarding the effect of TAP is inconclusive in patients with rheumatic mitral valve disease.

As RV is a crescent-shaped structure located in close proximity to the sternum, two-dimensional (2D) echocardiographic measurements of RV structure and function are often challenging. Especially for post-cardiac surgery patients, 2D-based assessment of longitudinal function may not be reflective of global RV function [6]. Three-dimensional (3D) echocardiography is well suited to evaluate RV volume and ejection fraction, and has been validated against cardiac magnetic resonance imaging [7]. Moreover, on the basis of full-volume data set, 3D speckle tracking echocardiography could provide comprehensive quantitative assessment of global RV myocardial function and avoid out-of-plane speckle motion in 2D imaging. Therefore, in this study, by the use of 3D echocardiography, we sought to evaluate the effect of TAP concomitant with MVR on RV remodeling and function for rheumatic mitral valve disease patients, and to compare the echocardiographic data of patients undergoing concomitant TAP with data of patients undergoing isolated MVR, preoperatively and at 1-year follow-up.

## Methods

### Study population

This research protocol was compliant to the ethical guidelines of the 1975 Declaration of Helsinki, and was approved by the institutional review board of West China hospital. Between March 2016 to June 2017, 211 patients with rheumatic mitral valve disease scheduled for MVR provided their willingness to participate in this study and signed informed consent. The diagnosis of rheumatic heart disease was according to the 2012 World Heart Federation criteria [8]. Patients had their clinical and echocardiographic assessment within 1 week before the operation. Thirteen patients were excluded for organic aortic valve or tricuspid valve (TV) disease, 10 for suboptimal echocardiographic window, 6 for coronary artery disease that needed revascularization, 1 for pacemaker implantation postoperatively. Five patients were lost to follow-up. At last, we enrolled 176 patients for the analysis.

All patients in this study underwent MVR, the use of mechanical mitral valve or bioprosthetic valve was according to patients’ age and anticoagulant plan, and the addition of Maze procedure for patients with atrial fibrillation was according to each patient’s informed preferences and the attending surgeon’s discretion. All patients with moderate and severe TR had concomitant TAP. If TR grade was less than moderate, the implementation of TAP was at the attending surgeon’s discretion. Tricuspid annular dilation (> 40 mm or > 21 mm/m^2^), atrial fibrillation, pulmonary hypertension and right heart failure were the main risk factors when considering whether less-than-moderate functional TR patients needed prophylactic TAP. However, some surgeons at our center would take rheumatic heart disease as a risk factor and perform prophylactic TAP for those with rheumatic mitral valve disease and less-than-moderate functional TR, irrespective of tricuspid annular dimension or pulmonary artery systolic pressure.

### Echocardiographic and clinical data collection

Comprehensive transthoracic echocardiographic examinations were performed using the Philips EPIQ 7C (Philips Medical Systems, Andover, Massachusettes, USA) with a 2-MHz to 4-MHz matrix-array transthoracic transducer, which could display both 2D and 3D full-volume images. Four consecutive cardiac cycles were recorded in patients with sinus rhythm and 6 cycles in atrial fibrillation. For patients with sinus rhythm, 3D full volume data set were obtained from 4 to 6 consecutive cardiac cycles during held respiration (frame rate ≥ 18 Hz). In patients with atrial fibrillation, high volume rate quantification mode was applied in the reconstruction of 3D full volume data. All the echocardiographic parameters were measure in 3 successive beats and then averaged.

LV volume and ejection fraction were calculated using Simpson’s biplane method from 2D imaging. Left and right atrial volume were calculated by the disk summation technique from apical 4-chamber view. The tricuspid annulus diameter was measured from RV-modified apical 4-chamber view in end-diastolic and was indexed to body surface area. 2D RV systolic function was assessed by tricuspid annular plane systolic excursion (TAPSE), RV fraction area change and tissue Doppler of the tricuspid annulus. Systolic RV pressure gradient was calculated by the modified Bernoulli equation and then pulmonary artery systolic pressure (PASP) was derived. According to current guidelines [9, 10], TR severity was qualitatively graded as 4 categories: no or trace (jet area < 1 cm^2^), mild (vena contracta < 3 mm, jet area < 5 cm^2^), moderate (3 mm ≤ vena contracta < 7 mm), and severe (vena contracta ≥7 mm).

**Fig. 1:**
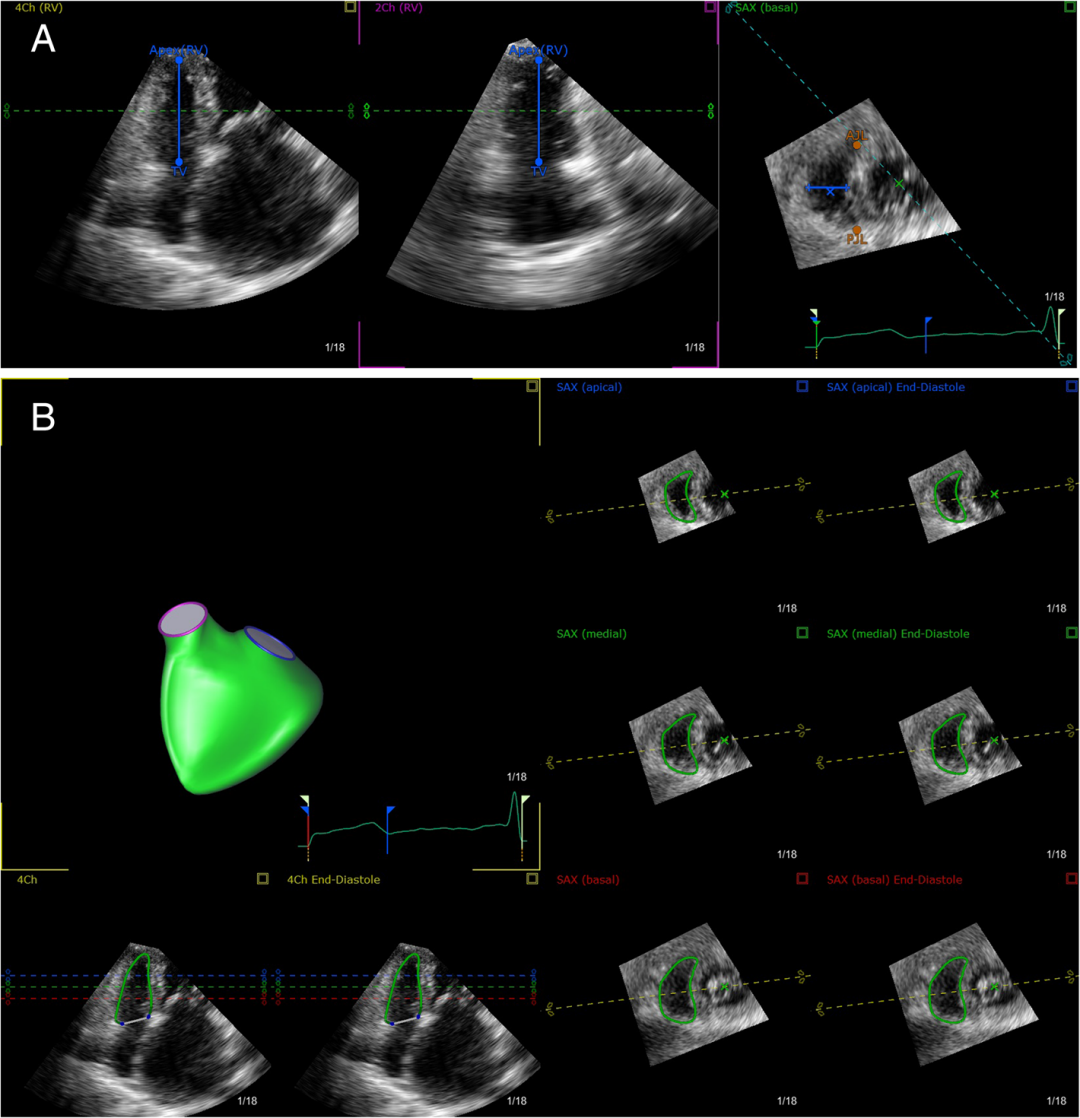
**a** Manual initialization of the right ventricular endocardium at specified points defined by the analysis software. **b** The automatically tracking of the right ventricular endocardium in long-axis and short-axis views

The 3D full volume images from RV-modified apical 4-chamber view were analyzed by the software of Tomtec Image Arena 4.0 on off-line Imaging workstation (TomTec Imaging Systems, Munich, Germany). After manual initialization of the RV endocardium at 8 points on the long axis and short axis views (Fig. 1, a), the software automatically traced the endocardium in the end-diastole and end-systole (Fig. 1, b). RV end-diastolic volume (RV EDV), RV end-systolic volume (RV ESV), RV ejection fraction (RVEF), RV longitudinal strain (RVLS) of free wall and septal wall were then calculated. The endocardial contours were manually adjusted to optimize the tracking of the myocardium. Negative strain values indicate myocardium contraction, reduced absolute values represented diminished contractile function. Reproducibility of 3D image analyses was determined in a random 10% of 3D images subgroup.

At baseline and during 1-year follow-up, symptoms were recorded and functional New York Heart Association (NYHA) class was assessed. Physical examination and electrocardiogram were performed before each echocardiographic assessment. Cardiopulmonary bypass time and other data of surgical procedure were extracted from medical records.

### Statistical analysis

Continuous variables were expressed as median and interquartile range, categorical variables were expressed as numbers and percentages. Comparison between 2 groups were performed by Mann-Whitney U test for continuous variables, and χ^2^ or Fisher’s exact test for categorical variables as appropriate. Cumulative incidence rates of survival were estimated by the Kaplan–Meier method and compared using the log-rank test. To compare the effect of with and without concomitant TAP on cardiac remodeling between groups across the preoperative and 1-year follow-up periods, 2-factor analysis of variance with repeated measures were conducted. Within groups, changes of each continuous variable at follow-up time were analyzed by Wilcoxon signed rank test, and changes of dichotomous variables were analyzed by McNemar test. A sensitive analysis was performed in patients with less-than-moderate functional TR who received concomitant TAP, we compared their pre- and post-operative data with patient who had less-than-moderate functional TR and isolated MVR.

To evaluate whether post-operative TR severity, RV remodeling and function were affected by multiple factors (including age, gender category, pre-operative PASP, concomitant Maze procedure, concomitant TAP), multivariate ordinal logistic regression was used to assess the risk factors for the severity of TR grade at 1-year follow-up, and multivariate linear regression was used to test the association between the abovementioned characteristics and percent change of postoperative RV EDV, RVEF, and RVLS.

Interobserver and intraobserver agreement for RV EDV, RVEF, RVLS of free wall and septal wall measurement were tested by Bland-Altman analysis (mean difference and standard deviation). Two independent blinded observers performed the offline analysis on 2 different days for 34 randomly selected images. Statistical analyses were conducted using the Statistical Package for Social Sciences release 25.0 (SPSS, Chicago, USA). All tests were 2-tailed, a *P* value < 0.05 was considered statistically significant.

## Results

### Baseline characteristics and the effect of TAP at follow-up

Among the 176 patients enrolled in this study, 6 patients were deceased within 6 months after the surgery, with 5 patients in the TAP group (3 patients deceased before discharge due to postoperative complication, 1 died of heart failure 1 month after the operation, 1 had sudden death 4 months after the operation, the cumulative survival rate was 96.1%), and 1 patient in the non-TAP group (due to stroke 6 months after the operation, the cumulative survival rate was 97.9%). No significant difference in all-cause mortality between the two groups was found (log-rank *P* = 0.57). Finally, 170 patients with complete 1-year postoperative clinical records and echocardiographic data were assessed (concomitant TAP group, *n* = 124; non-TAP group, *n* = 46). One hundred thirty-three patients had mechanical mitral valve and 37 had bioprosthetic valve. Concomitant tricuspid repair by DeVega annuloplasty was performed in 28 patients, and ring annuloplasty in 96 patients (Cosgrove-Edwards annuloplasty band with size of 26 mm, 28 mm, 30 mm and 32 mm). Table 1 demonstrated baseline characteristics and the operative information of the two groups. Compared with patients had isolated MVR, patients undergoing concomitant TAP were older, had smaller body size, worse cardiac function class, and higher prevalence of atrial fibrillation. With regard to echocardiographic data, preoperative TR grade were significantly greater in the TAP group, this group also had larger right heart volume, more dilated tricuspid annulus, and more decreased LV and RV function compared with non-TAP group at baseline (all *P* <  0.001). While patients in the isolated MVR group all had less-than-moderate TR. Cardiopulmonary bypass time were longer in TAP group than the non-TAP group, and Maze procedure was more frequently performed in the TAP group.

**Table 1:** Preoperative characteristics of patients with and without concomitant tricuspid annuloplasty

Variables	TAP group (***n*** = 124)	Non-TAP group (***n*** = 46)	***P*** value
Age, y	53.0 (47.0, 62.0)	50.0 (44.0, 59.0)	0.03
Female	98 (79%)	33 (72%)	0.32
Body surface area, m^2^	1.53 (1.45, 1.62)	1.59 (1.49, 1.66)	0.04
NYHA functional class III-IV	92 (74%)	22 (48%)	0.001
Atrial fibrillation	99 (80%)	16 (35%)	< 0.001
Echocardiographic data			
LV end-diastolic volume, ml	102.0 (86.3, 127.8)	100.5 (88.8, 120.0)	0.99
LV ejection fraction, %	61.0 (54.0, 66.0)	67.0 (60.0, 68.3)	< 0.001
Left atrial volume, ml	135.0 (99.0, 173.0)	119.5 (89.2, 138.0)	0.03
Right atrial volume, ml	51.1 (39.7, 67.0)	33.5 (25.8, 44.0)	< 0.001
TR grade			
None to trace	3 (3%)	17 (37%)	< 0.001
Mild	29 (23%)	29 (63%)	< 0.001
Moderate	57 (46%)	0	< 0.001
Severe	35 (28%)	0	< 0.001
TV annulus diameter, mm	34.0 (31.0, 36.8)	28.0 (27.0, 31.0)	< 0.001
Indexed TV annulus diameter, mm/m^2^	22.3 (20.6, 23.8)	18.2 (17.2, 19.6)	< 0.001
Pulmonary artery systolic pressure, mmHg	38.6 (33.0, 47.8)	29.8 (24.4, 38.0)	< 0.001
RV inlet dimension, mm	36.3 (32.9, 39.3)	32.9 (31.3, 37.0)	< 0.001
TAPSE, mm	11.9 (9.1, 15.2)	14.4 (10.4, 17.4)	0.01
RV fractional area change, %	40.2 (34.0, 44.9)	41.7 (36.0, 47.8)	0.09
Tissue Doppler of the tricuspid annulus, cm/s	9.2 (7.9, 10.7)	9.7 (8.2, 11.8)	0.15
RV end-diastolic volume, ml	51.3 (43.4, 64.6)	41.3 (35.8, 46.1)	< 0.001
RV ejection fraction, %	32.6 (28.6, 35.2)	40.9 (36.6, 46.1)	< 0.001
RVLS of free wall, %	−15.5 (−18.6, −13.4)	−22.0 (−24.3, −18.7)	< 0.001
RVLS of septal wall, %	−10.2 (−11.9, −8.6)	−12.3 (− 13.7, −10.9)	< 0.001
Operative data			
Concomitant MAZE procedures	59 (48%)	2 (4%)	< 0.001
Mechanical mitral prosthesis	94 (76%)	39 (85%)	0.21
Cardiopulmonary bypass time, min	100.0 (84.0, 126.8)	78.5 (66.8, 99.2)	< 0.001
MV lesion			
Stenosis	79 (64%)	34 (74%)	0.21
Regurgitation	17 (14%)	6 (13%)	0.91
Mixed	28 (23%)	6 (13%)	0.17

*LV* left ventricle, *MV* mitral valve, *NYHA* New York Heart Association, *RV* right ventricle, *RVLS* right ventricular longitudinal strain, *TAP* tricuspid annuloplasty, *TAPSE* tricuspid annular plane systolic excursion, *TR* tricuspid regurgitation, *TV* tricuspid valve

Data are expressed as median (interquartile range) or *n* (%)

**Fig. 2:**
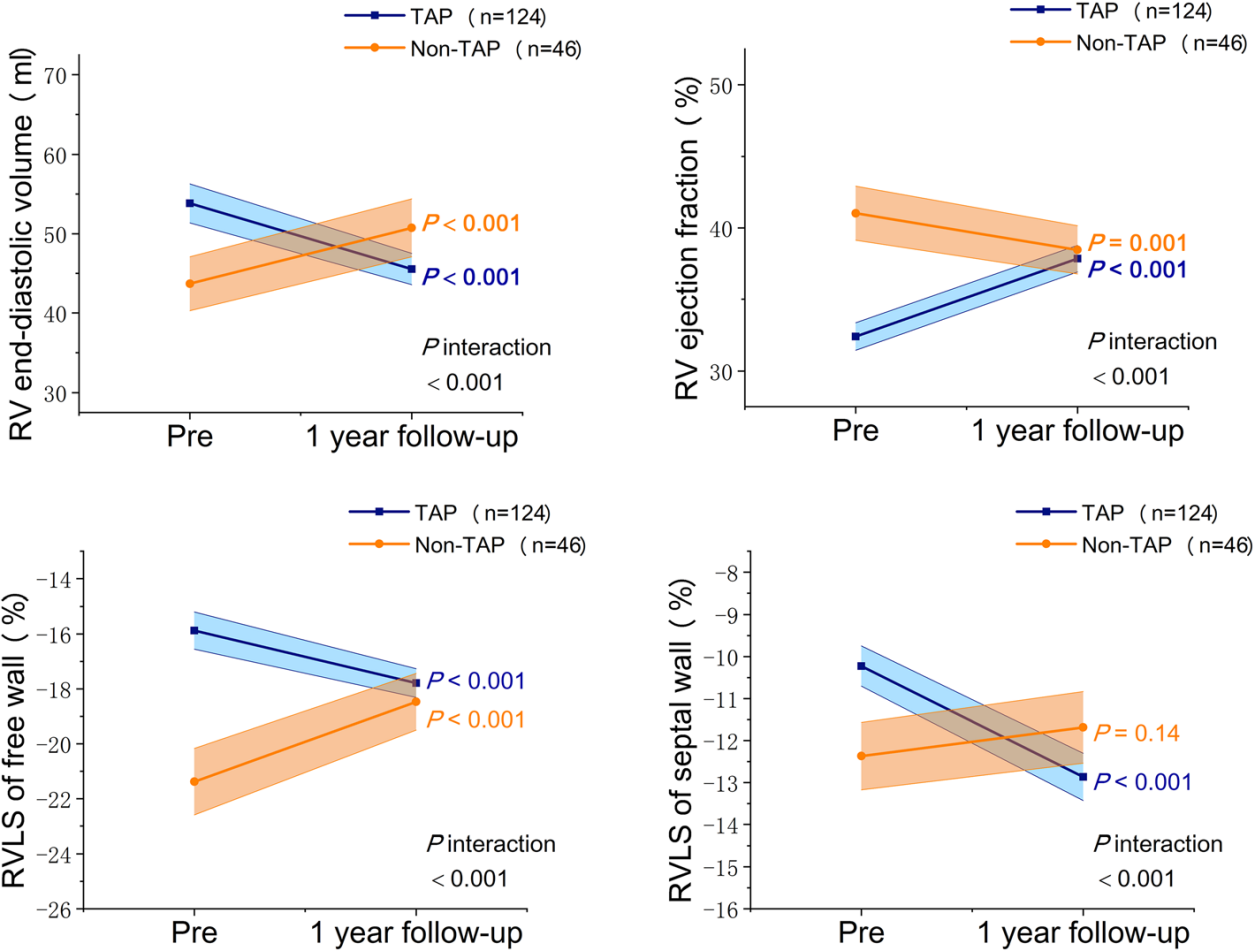
The longitudinal changes and interactions between groups and time of three-dimensional right ventricular echocardiographic parameters, according to patients undergoing mitral valve surgery with and without concomitant tricuspid annuloplasty. The *P* values of longitudinal comparison were showed in colors, and P values of interaction by 2-factor analysis of variance with repeated measures were showed in black. Pre, preoperative; RV, right ventricle; RVLS, right ventricular longitudinal strain; TAP, tricuspid annuloplasty

Changes of clinical and echocardiographic data at 1-year follow-up were summarized in Table 2, and the results of paired pre- and post-operative comparison as well as the interaction between groups and time were also showed. After surgery, the prevalence of atrial fibrillation was only significantly decreased in the TAP group. TR severity of the TAP group relieved at 1-year follow-up. However, we observed TR progression in the non-TAP group, in which 24% patients developed moderate and severe TR at follow-up. In terms of quantitative echocardiographic parameter, we considered the significant interaction between groups and time as the effect of concomitant TAP. The left heart volume and 2D parameters of RV did not show significant interaction, while estimated PASP and 3D RV parameters including RV EDV, RVEF and RVLS all showed significant interaction (all *P* <  0.001). Longitudinal changes of the 3D RV parameters were also demonstrated in Fig. 2. TAP group had worse baseline RV parameters, but during 1-year follow-up, RV EDV reduced, RVEF and RV longitudinal strain improved. Whereas patients in non-TAP group had dilated RV, deteriorated RVEF and RVLS of free wall at follow-up. So that at 1 year after the surgery, there was no difference of RVEF or RVLS between the groups, while non-TAP group had slightly larger RV EDV (*P* = 0.01).

**Table 2:** Clinical and echocardiographic data at 1-year follow-up

	One year follow-up data			Compared with preoperative data		Two-way ANOVA
	TAP (***n*** = 124)	Non –TAP (***n*** = 46)	***P*** value	TAP (***n*** = 124)	Non –TAP (***n*** = 46)	Interaction
NYHA functional class III-IV	21 (17%)	1 (2%)	0.009	< 0.001	< 0.001	–
Atrial fibrillation	54 (44%)	11 (24%)	0.02	< 0.001	0.13	–
Echocardiographic data						
TR grade						
None to trace	76 (61%)	11 (24%)	< 0.001	< 0.001	0.18	–
Mild	33 (27%)	24 (52%)	0.002	0.67	0.35	–
Moderate	14 (11%)	10 (22%)	0.09	< 0.001	0.002	–
Severe	1 (1%)	1 (2%)	0.47	< 0.001	1.00	–
LV end-diastolic volume, ml	102.0 (85.3, 112.0)	104.0 (90.5, 116.0)	0.42	0.06	0.54	0.76
LV ejection fraction, %	60.0 (58.0, 63.0)	61.0 (58.0, 62.3)	0.33	0.67	0.003	0.03
Left atrial volume, ml	86.9 (66.4, 114.0)	83.8 (57.8, 106.5)	0.15	< 0.001	< 0.001	0.63
Right atrial volume, ml	38.4 (31.7, 52.0)	38.6 (29.5, 44.6)	0.20	< 0.001	0.08	< 0.001
Pulmonary artery systolic pressure, mmHg	24.3 (20.7, 29.8)	24.7 (21.2, 28.1)	0.81	< 0.001	< 0.001	< 0.001
RV inlet dimension, mm	35.7 (32.9, 38.5)	35.8 (33.1, 37.3)	0.52	0.13	0.006	0.13
TAPSE, mm	12.3 (9.4, 14.3)	13.3 (11.2, 15.5)	0.03	0.42	0.06	0.27
RV fractional area change, %	42.3 (37.5, 46.6)	42.8 (38.4, 47.9)	0.35	0.005	0.16	0.44
Tissue Doppler of the tricuspid annulus, cm/s	8.6 (7.6, 9.4)	8.5 (7.9, 9.5)	0.77	< 0.001	< 0.001	0.24
RV end-diastolic volume, ml	44.5 (36.8, 51.4)	48.0 (42.9, 57.4)	0.01	< 0.001	< 0.001	< 0.001
RV ejection fraction, %	38.1 (34.5, 42.0)	38.6 (34.3, 42.4)	0.72	< 0.001	0.001	< 0.001
RVLS of free wall, %	−18.2 (−19.8, −15.9)	−19.2 (−20.7, −15.7)	0.32	< 0.001	< 0.001	< 0.001
RVLS of septal wall, %	−12.1 (− 15.8, −10.6)	−11.4 (−13.5, −8.9)	0.06	< 0.001	0.14	< 0.001

*LV* left ventricle, *NYHA* New York Heart Association, *RV* right ventricle, *RVLS* right ventricular longitudinal strain, *TAP* tricuspid annuloplasty, *TR* tricuspid regurgitation

Data are expressed as median (interquartile range) or *n* (%)

**Fig. 3:**
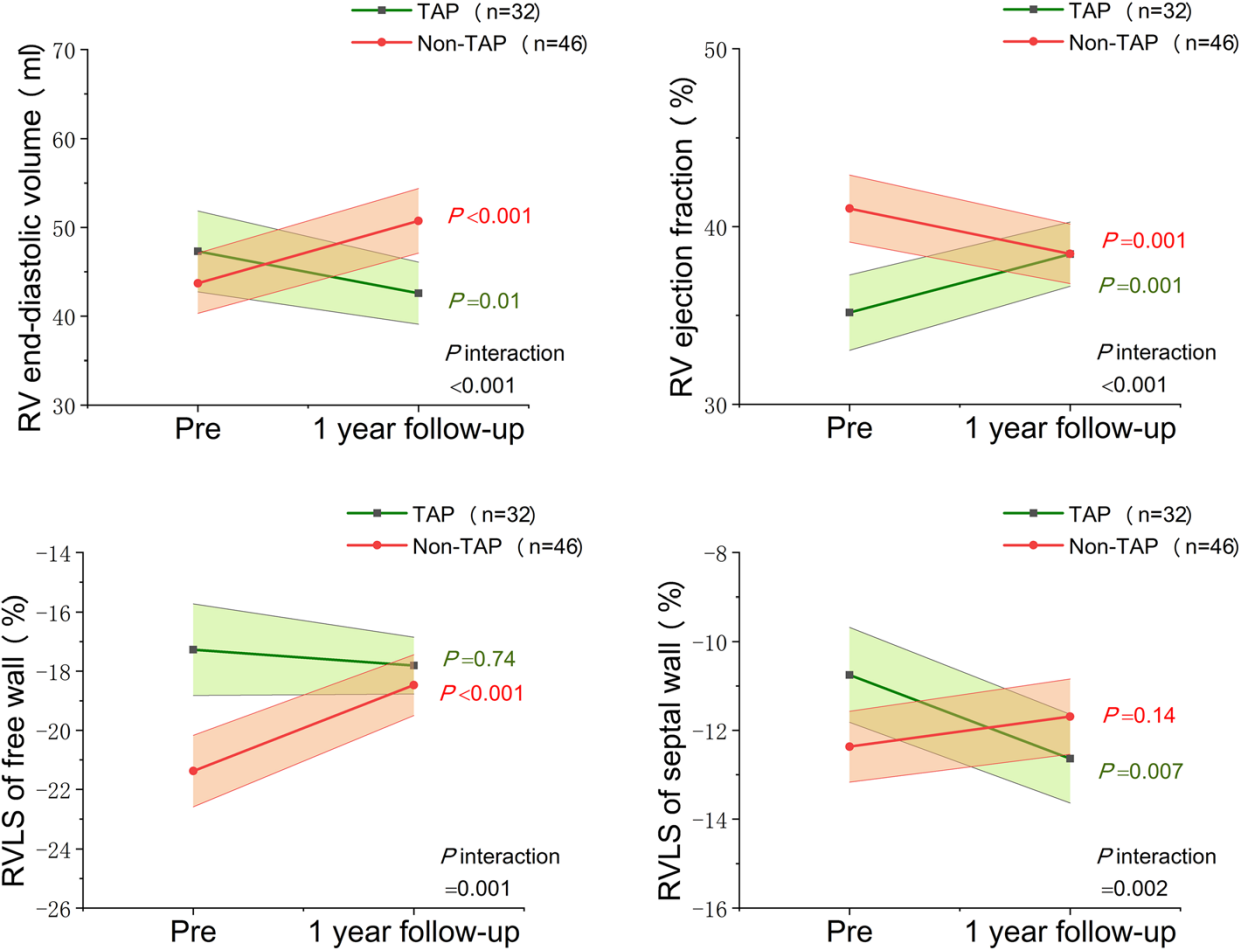
The longitudinal changes and interactions between groups and time of three-dimensional right ventricular echocardiographic parameters, according to patients with less than moderate TR undergoing mitral valve surgery with and without concomitant tricuspid annuloplasty. The P values of longitudinal comparison were showed in colors, and P values of interaction by 2-factor analysis of variance with repeated measures were showed in black. Pre, preoperative; RV, right ventricle; RVLS, right ventricular longitudinal strain; TAP, tricuspid annuloplasty

The results of sensitive analysis were presented in Fig. 3 and additional files. In patients with preoperatively less-than-moderate functional TR, 41% received prophylactic TAP concomitant with MVR (*n* = 32), the other 59% received isolated MVR (*n* = 46). Additional Table 1 demonstrated their baseline characteristics. TAP group was older, with more atrial fibrillation, and functional TR grade were more severe than the non-TAP group, also the preoperative RV function were worse in TAP group. But preoperative RV EDV was comparable between the two groups. Similar to the main analysis, at 1-year follow-up, TR severity was worse in the non-TAP group. RV EDV, RVEF and RVLS of the septal wall demonstrated different changes in reverse direction according to group, TAP group had reduced RV EDV, increased RVEF, and improved RVLS of septal wall. On the contrary, non-TAP group showed dilated RV EDV and deteriorated RV function. For patients with less-than-moderate TR, 3D parameters of RV EDV, RVEF, RVLS of free wall and septal wall showed significant interaction between surgery approaches and time. (Fig. 3 and additional Table 2).

### Predictors of postoperative TR severity, RV remodeling and function

Table 3 showed the univariate and multivariate ordinal logistic regression analysis according to the grade of TR severity at 1-year follow-up. By univariate regression, increasing age was a risk factor for postoperative TR deterioration, while Maze procedure and TAP concomitant with MVR were protective factors for patients from TR worsening. Post-operative atrial fibrillation showed no associated with TR grade. In multivariate analysis, concomitant TAP remained as the independent predictor for the relief of postoperative TR (Odds Ratio: 0.45; 95% Confidence Interval: 0.22–0.91; *P* = 0.025). Predictors of longitudinal changes of RV volume and function by multivariate linear regression were demonstrated in Table 4. TAP was the main independent predictor of RV volume decrease at 1-year follow-up (R^2^ = 0.41, Standardized β = − 0.557, *P* <  0.001). TAP was also the independent predictor for RVEF increment (R^2^ = 0.29, Standardized β = 0.364, *P* <  0.001), as well as for improvement of RVLS of the free wall (R^2^ = 0.28, Standardized β = 0.206, *P* = 0.01) and septal wall (R^2^ = 0.14, Standardized β = 0.28, *P* = 0.001). In general, TAP was closely related to the RV reverse remodeling and functional improvement at 1-year follow-up. Meanwhile, concomitant Maze procedure was the independent predictor of the postoperative RVEF and RV strain improvement, but no significant correlation was found between Maze procedure and change of RV EDV at 1-year follow-up.

**Table 3:** Predictors of post-operative TR severity at 1-year follow-up by ordinal logistic regression

	Univariate		Multivariate	
	***P*** value	OR (95% CI)	***P*** value	OR (95% CI)
Age	0.02	1.03 (1.01–1.07)	0.17	1.02 (0.99–1.05)
Female	0.89	1.05 (0.53–2.08)	–	–
Pre-operative PASP	0.88	0.99 (0.98–1.02)	–	–
Post-operative atrial fibrillation	0.14	1.56 (0.87–2.82)	–	–
Concomitant Maze procedure	< 0.001	0.21 (0.10–0.41)	0.001	0.29 (0.14–0.61)
Concomitant TAP	< 0.001	0.27 (0.14–0.52)	0.025	0.45 (0.22–0.91)

*CI* confidence interval, *OR* odds ratio, *PASP* Pulmonary artery systolic pressure, *TAP* tricuspid annuloplasty, *TR* tricuspid regurgitation

**Table 4:** Multivariate linear regression analysis of the predictors for post-operative right ventricular remodeling and function in rheumatic valve patients undergoing mitral valve replacement

Dependent variables	Independent variables	Standardized β	***P*** value
Percent change of RV EDV at 1-year follow-up	Age	0.004	0.95
	Female	0.045	0.45
	Pre-operative PASP	0.163	0.01
	Concomitant Maze procedure	−0.027	0.69
	Concomitant TAP	−0.557	< 0.001
Percent change of RVEF at 1-year follow-up	Age	0.102	0.14
	Female	0.004	0.95
	Pre-operative PASP	0.026	0.71
	Concomitant Maze procedure	0.212	0.004
	Concomitant TAP	0.364	< 0.001
Percent change of free wall RVLS at 1-year follow-up	Age	0.149	0.03
	Female	−0.051	0.45
	Pre-operative PASP	0.059	0.41
	Concomitant Maze procedure	0.342	< 0.001
	Concomitant TAP	0.206	0.01
Percent change of septal wall RVLS at 1-year follow-up	Age	0.01	0.90
	Female	−0.02	0.74
	Pre-operative PASP	0.10	0.20
	Concomitant Maze procedure	0.11	0.18
	Concomitant TAP	0.28	0.001

*EDV* end diastolic volume, *PASP* Pulmonary artery systolic pressure, *RV* right ventricle, *RVEF* right ventricular ejection fraction, *RVLS* right ventricular longitudinal strain, *TAP* tricuspid annuloplasty, *TR* tricuspid regurgitation

### Reproducibility

Inter-observer reproducibility by Bland-Altman analysis demonstrated good agreement: mean difference (standard deviation) was − 0.06 (±1.59) for RV end-diastolic volume, − 0.21 (±1.59) for RVEF, 0.35 (±1.34) and 0.16 (±1.94) for RVLS of the free and septal wall. Also good agreement was seen in intra-observer reproducibility: 0.01 (±1.22) for RV end-diastolic volume, 0.18 (±1.05) for RVEF, 0.24 (±0.79) and 0.21 (±0.85) for RVLS of the free and septal wall.

## Discussion

The major findings of the present study were the following: (1) Routine TAP at the time of MVR in rheumatic mitral valve patients could relieve residual functional TR, promote RV reverse remodeling and improve RV function in patients with worse baseline characteristics; (2) RV dilation and functional deterioration were observed in patients with isolated MVR at 1-year follow-up. These findings support the approach to perform TAP concomitant with MVR in rheumatic mitral valve disease patients. Prophylactic TAP could prevent RV dilation and functional deterioration postoperatively.

Patients with mitral valve disease often have functional TR, concomitant TAP with left heart operation is an effective treatment to reduce TR grade and improve RV function. Since increasing TR severity was reported to be independently associated with worse long-term survival, and functional TR could progress even after left heart valve surgery [11, 12], to repair severe TR concomitant with left heart surgery is now the routine practice. Also, patients with risk factors such as tricuspid annular dilation and right heart failure could benefit from concomitant TAP even in the absence of severe TR. Moreover, previous studies reported that moderate TR per se was not benign, and concomitant tricuspid repair for those patients resulted in RV reverse remodeling, preventing postoperative TR progression [5, 13, 14]. Our findings resonated with the previous studies, in addition, 3D echocardiography and speckle tracking could provide a more detailed description of the RV. As TR severity is preload and afterload dependent and often inconstant, merely the TR severity or annular dimension might not be enough to identify patients at risk, especially when under anesthesia [15]. 2D parameters of RV function were less sensitive to detect post-operative changes compared with 3D measurements in our patients. Measurement of RV geometry and function by 3D speckle tracking could provide complementary information for follow-up evaluation, as well as for the decision-making process when considering TAP concomitant with mitral valve surgery. Besides, Ye et al. reported that RV morphology and function were more associated with postoperative prognosis than functional TR grade [16]. Kammerlander et al. also demonstrated that RV dysfunction is independently correlated with survival late after left heart valve procedure [17]. Our observational study showed that TAP improved postoperative RV function, but no difference of mortality between TAP and non-TAP groups was found during 1-year follow-up. Long-term follow-up is still in progress, and 3D echocardiography remains a convenient method for preoperative and follow-up assessment of RV morphology and function.

The postoperative deterioration of RV volume and function in patients with isolated MVR is a concern for surgeon to perform prophylactic TAP for less-than-moderate functional TR. Sakata et al. had reported that in patients with moderate and mild TR who had no concomitant TAP, worsening of RV systolic function and dilation of RV diameter were prominent at 6-month to 1-year follow-up, while RV diastolic function was preserved [18, 19]. Similarly, Chikwe et al. demonstrated that RV dysfunction was prominent at discharge and around 1-year follow-up for patients undergoing mitral valve surgery [5]. Nevertheless, a random trial by Pettinari et al. revealed that, in patients with less-than-severe functional TR, prophylactic TAP irrespective of annular dilatation during mitral valve surgery could prevent significant postoperative TR, but had no effect on RV function and remodeling at 5-year follow-up [20]. The extrapolation of this randomized controlled trial should be cautious, as almost 60% of the patients had degenerative mitral disease, rheumatic valve disease patients might be under-represented in the trial. It had been observed that RV dilation and TR progression were rare after successful repair of degenerative mitral regurgitation without TAP [21]. However, in our patients of rheumatic mitral valve disease who had valve replacement rather than repair, postoperative RV dilation were seen in those with less-than-moderate preoperative TR receiving isolated MVR. We inferred that differences in the outcomes among our study and the previous studies probably due to the different etiologies of valvular disease. Rheumatic mitral valve patients are usually female, with higher prevalence of atrial fibrillation, having elevated PASP and lower body mass index compared with patients of degenerative mitral disease, which were risk factors of TR progression [22–24]. Even though MVR relieved LA overload and decreased pulmonary pressure, deterioration of TR grade and RV performance was still observed in our patients with mitral valve replacement. Besides the above risk factors, the possible cause might be attributed to the hypoxia during the operation, and prosthetic mitral valve might affect the shape and function of the tricuspid apparatus. Our result implied that prophylactic TAP could be considered for rheumatic mitral valve disease patients with less-than-moderate functional TR.

Whether adding TAP to left heart surgery for mild or trace TR could improve postoperative survival remains to be clarified. Kim et al. found that, in rheumatic mitral valve patients, risk of death and congestive heart failure were similar between TAP and non-TAP group for mild-to-moderate TR [25]. In another study of 959 patients with mild-to-moderate TR, concomitant TAP was not associated with better clinical outcomes, but resulted in reduced postoperative TR grade [26]. In the research by Lee et al. of patients with less-than-moderate TR undergoing MVR, TAP showed a tendency to prevent TV-related events in a median follow-up duration of 8 years, notably 93% of their patients had rheumatic etiology [27]. Besides, positive effect of prophylactic TAP on valve-related events was found in rheumatic valve disease patients with less-than-moderate TR undergoing double valve replacement [28]. Whether patients with more rheumatic burden could benefit more from prophylactic TAP concomitant with left heart surgery was a debated topic. Although concomitant TAP had positive effect on RV geometry and function in our rheumatic patients, the long-term clinical effect still need to be assessed. Even with less-than-moderate TR, rheumatic valvular disease patients receiving concomitant TAP were older, with atrial fibrillation, and decreased RV function compared with the isolated MVR group. Nevertheless, concomitant TAP improved RV geometry and function at 1-year follow-up in our patients with worse baseline risk factor. Considering the safety of performing additional TAP concomitant with left heart surgery and the risk of reoperation for severe TR [29], we incline to suggest prophylactic TAP concomitant with MVR for rheumatic mitral valve disease to prevent RV dilation and TR progression at follow-up.

There were several limitations in our study. First, as its observational nature, patients were treated by several surgeons in whom different standards for operation and different surgical technique existed, some patients with less-than-moderate TR in our study had no guideline-recommended indication of concomitant TAP, for whom prophylactic TAP was based on the attending surgeon’s discretion. This selection bias and the potential confounder brought by different surgeons was inevitable. Second, the power of tests could be affected as the sample size was limited. Due to the limited sample size of patients with less-than-moderate TR, propensity score matching could not achieve sufficient pairs of patients from concomitant TAP and non-TAP group, and the comparison was underpowered. The effect of concomitant TAP in rheumatic valvular disease patients with less-than-moderate TR need to be tested by randomized control trial or large observational cohort. Third, there was no analysis of the RV geometry and function immediately postoperative, thus the trends of RV remodeling after the surgery were incomplete. Another limitation was that, due to the short-term follow-up of 1 year, we were not able to detect the long-term clinical outcome of concomitant TAP, or whether postoperative RV reverse remodeling could reduce valve-related adverse cardiac events. Further studies with large sample size and long-term follow-up is required.

## Conclusion

In this study focused exclusively on rheumatic mitral valve disease patients, we found that concomitant TAP with MVR had positive effect on postoperative RV geometry and function, independent of concomitant Maze procedure. Whereas patients with isolated MVR had RV dilation and deterioration of RV function at 1-year follow-up. Our results support that rheumatic mitral valve patients undergoing MVR could benefit from concomitant surgery for functional TR.

## Supplementary Information

**Additional file 1: Additional Table 1.** Preoperative characteristics of patients with less-than-moderate functional tricuspid regurgitation. **Additional Table 2.** Follow-up data in patients with less-than-moderate functional tricuspid regurgitation

## Data Availability

All data generated and analyzed in this study is available from the corresponding author on reasonable request.

## Abbreviations

2D: Two-dimensional
3D: Three-dimensional
EDV: End-diastolic volume
EF: Ejection fraction
ESV: End-systolic volume
LV: Left ventricle
MV: Mitral valve
MVR: Mitral valve replacement
NYHA: New York Heart Association
RV: Right ventricle
RVLS: Right ventricular longitudinal strain
TAP: Tricuspid annuloplasty
TR: Tricuspid regurgitation
TV: Tricuspid valve

## References

[1] Nishimura RA, Otto CM, Bonow RO, Carabello BA, Erwin JP, Guyton RA, O’Gara PT, Ruiz CE, Skubas NJ, Sorajja P (2014). 2014 AHA/ACC guideline for the management of patients with valvular heart disease: a report of the American College of Cardiology/American Heart Association task force on practice guidelines. J Am Coll Cardiol.

[2] Baumgartner H, Falk V, Bax JJ, De Bonis M, Hamm C, Holm PJ, Iung B, Lancellotti P, Lansac E, Munoz DR (2017). 2017 ESC/EACTS guidelines for the management of valvular heart disease. Eur Heart J.

[3] Zadok OIB, Sagie A, Vaturi M, Shapira Y, Schwartzenberg S, Kuznitz I, Shochat T, Bental T, Yedidya I, Aravot D (2019). Long-term outcomes after mitral valve replacement and tricuspid annuloplasty in rheumatic patients. Ann Thorac Surg.

[4] Bertrand PB, Koppers G, Verbrugge FH, Mullens W, Vandervoort P, Dion R, Verhaert D (2014). Tricuspid annuloplasty concomitant with mitral valve surgery: effects on right ventricular remodeling. J Thorac Cardiovasc Surg.

[5] Chikwe J, Itagaki S, Anyanwu A, Adams DH (2015). Impact of concomitant tricuspid annuloplasty on tricuspid regurgitation, right ventricular function, and pulmonary artery hypertension after repair of mitral valve prolapse. J Am Coll Cardiol.

[6] Venkatachalam S, Wu G, Ahmad M (2017). Echocardiographic assessment of the right ventricle in the current era: application in clinical practice. Echocardiography..

[7] Sugeng L, Mor-Avi V, Weinert L, Niel J, Ebner C, Steringer-Mascherbauer R, Bartolles R, Baumann R, Schummers G, Lang RM (2010). Multimodality comparison of quantitative volumetric analysis of the right ventricle. JACC Cardiovasc Imaging.

[8] Reményi B, Wilson N, Steer A, Ferreira B, Kado J, Kumar K, Lawrenson J, Maguire G, Marijon E, Mirabel M (2012). World heart federation criteria for echocardiographic diagnosis of rheumatic heart disease—an evidence-based guideline. Nat Rev Cardiol.

[9] Lancellotti P, Tribouilloy C, Hagendorff A, Popescu BA, Edvardsen T, Pierard LA, Badano L, Zamorano JL (2013). Recommendations for the echocardiographic assessment of native valvular regurgitation: an executive summary from the European Association of Cardiovascular Imaging. Eur Heart J Cardiovasc Imaging.

[10] Zoghbi WA, Adams D, Bonow RO, Enriquez-Sarano M, Foster E, Grayburn PA, Hahn RT, Han Y, Hung J, Lang RM (2017). Recommendations for noninvasive evaluation of native valvular regurgitation: a report from the American Society of Echocardiography developed in collaboration with the Society for Cardiovascular Magnetic Resonance. J Am Soc Echocardiogr.

[11] Nath J, Foster E, Heidenreich PA (2004). Impact of tricuspid regurgitation on long-term survival. J Am Coll Cardiol.

[12] Dreyfus GD, Corbi PJ, Chan KJ, Bahrami T (2005). Secondary tricuspid regurgitation or dilatation: which should be the criteria for surgical repair?. Ann Thorac Surg.

[13] Fawzy HF, Morsy AA, Serag AR, Elkahwagy MS, Sami G, Wahby EA, Arafat AA (2020). Should moderate functional tricuspid regurgitation be repaired during surgery for rheumatic mitral valve disease?. Heart Lung Circ.

[14] Kara I, Koksal C, Erkin A, Sacli H, Demirtas M, Percin B, Diler MS, Kirali K (2015). Outcomes of mild to moderate functional tricuspid regurgitation in patients undergoing mitral valve operations: a meta-analysis of 2,488 patients. Ann Thorac Surg.

[15] David TE, David CM, Manlhiot C (2018). Tricuspid annulus diameter does not predict the development of tricuspid regurgitation after mitral valve repair for mitral regurgitation due to degenerative diseases. J Thorac Cardiovasc Surg.

[16] Ye Y, Desai R, Abello LMV, Rajeswaran J, Klein AL, Blackstone EH, Pettersson GB (2014). Effects of right ventricular morphology and function on outcomes of patients with degenerative mitral valve disease. J Thorac Cardiovasc Surg.

[17] Kammerlander AA, Marzluf BA, Graf A, Bachmann A, Kocher A, Bonderman D, Mascherbauer J (2014). Right ventricular dysfunction, but not tricuspid regurgitation, is associated with outcome late after left heart valve procedure. J Am Coll Cardiol.

[18] Sakata T, Mogi K, Sakurai M, Nomura A, Fujii M, Kaneyuki D, Matsumiya G, Takahara Y (2018). Effect of tricuspid annuloplasty concomitant with left heart surgery on right heart geometry and function. J Thorac Cardiovasc Surg.

[19] Sakata T, Mogi K, Sakurai M, Tani K, Hashimoto M, Shiko Y, Kawasaki Y, Matsumiya G, Takahara Y (2020). Impact of tricuspid annuloplasty on postoperative changes in the right ventricular systolic and diastolic function: a retrospective cohort study. J Card Surg.

[20] Pettinari M, De Kerchove L, Lazam S, Pasquet A, Gerber B, Vanoverschelde JL, El-Khoury G (2019). Mid-term results of a randomized trial of tricuspid annuloplasty for less-than-severe functional tricuspid regurgitation at the time of mitral valve surgery. Eur J Cardiothorac Surg.

[21] Sordelli C, Lancellotti P, Carlomagno G, Di Giannuario G, Alati E, De Bonis M, Alfieri O, La Canna G (2016). Tricuspid annular size and regurgitation progression after surgical repair for degenerative mitral regurgitation. Am J Cardiol.

[22] Ong K, Yu G, Jue J (2014). Prevalence and spectrum of conditions associated with severe tricuspid regurgitation. Echocardiography..

[23] Wang G, Sun Z, Xia J, Deng Y, Chen J, Su G (2008). Predictors of secondary tricuspid regurgitation after left-sided valve replacement. Surg Today.

[24] Chen Y, Liu JH, Chan D, Sit KY, Wong CK, Ho KL, Ho LM, Zhen Z, Lam YM, Lau CP (2016). Prevalence, predictors and clinical outcome of residual pulmonary hypertension following tricuspid annuloplasty. J Am Heart Assoc.

[25] Kim JB, Yoo DG, Kim GS, Song H, Jung SH, Choo SJ, Chung CH, Lee JW (2012). Mild-to-moderate functional tricuspid regurgitation in patients undergoing valve replacement for rheumatic mitral disease: the influence of tricuspid valve repair on clinical and echocardiographic outcomes. Heart..

[26] Ro SK, Kim JB, Jung SH, Choo SJ, Chung CH, Lee JW (2013). Mild-to-moderate functional tricuspid regurgitation in patients undergoing mitral valve surgery. J Thorac Cardiovasc Surg.

[27] Lee H, Sung K, Kim WS, Lee YT, Park SJ, Carriere KC, Park PW (2016). Clinical and hemodynamic influences of prophylactic tricuspid annuloplasty in mechanical mitral valve replacement. J Thorac Cardiovasc Surg.

[28] Jeong DS, Shim MS, Sung K, Kim WS, Lee YT, Park PW (2015). Prophylactic tricuspid annuloplasty in patients undergoing double valve replacement. J Heart Valve Dis.

[29] McCarthy PM, Bhudia SK, Rajeswaran J, Hoercher KJ, Lytle BW, Cosgrove DM, Blackstone EH (2004). Tricuspid valve repair: durability and risk factors for failure. J Thorac Cardiovasc Surg.

