# Supplementary material for: Impact of concomitant tricuspid annuloplasty on right ventricular remodeling in patients with rheumatic mitral valve disease
Source: Cardiovasc Ultrasound. 2021 Mar 4;19:16. doi: 10.1186/s12947-021-00245-2 (PMC7934510; doi:10.1186/s12947-021-00245-2)
Supplement: Supplementary file 1 — Additional file 1: Additional Table 1. Preoperative characteristics of patients with less-than-moderate functional tricuspid regurgitation. Additional Table 2. Follow-up data in patients with less-than-moderate functional tricuspid regurgitation [file 12947_2021_245_MOESM1_ESM.docx]

**Additional Table 1.** Preoperative characteristics of patients with less-than-moderate functional tricuspid regurgitation

| **Variables** | **TAP group (n=32)** | |  | **Non-TAP group (n=46)** | ***P* value** |
| --- | --- | --- | --- | --- | --- |
| Age, y | 54.0 (47.3, 63.0) |  | | 50.0 (44.0, 59.0) | 0.04 |
| Female | 26 (81%) |  | | 33 (72%) | 0.43 |
| Body surface area, m^2^ | 1.54 (1.42, 1.58) |  | | 1.59 (1.49, 1.66) | 0.05 |
| NYHA functional class III-IV | 20 (63%) |  | | 22 (48%) | 0.25 |
| Atrial fibrillation | 19 (59%) |  | | 16 (35%) | 0.04 |
| Echocardiographic data |  |  | |  |  |
| LV end-diastolic volume, ml | 100.0 (77.5, 127.5) |  | | 100.5 (88.8, 120.0) | 0.55 |
| LV ejection fraction, % | 62.5 (56.0, 68.0) |  | | 67.0 (60.0, 68.3) | 0.11 |
| Left atrial volume, ml | 120.5 (76.1, 163.3) |  | | 119.5 (89.2,138.0) | 0.96 |
| Right atrial volume, ml | 41.4 (30.3, 47.1) |  | | 33.5 (25.8, 44.0) | 0.16 |
| TR grade |  |  | |  |  |
| None to trace | 3 (9%) |  | | 17 (37%) | 0.008 |
| Mild | 29 (91%) |  | | 29 (63%) | 0.008 |
| TV annulus diameter, mm | 31.0 (28.0, 34.0) |  | | 28.0 (27.0, 31.0) | 0.02 |
| Indexed TV annulus diameter, mm/m^2^ | 20.6 (18.9, 21.8) |  | | 18.2 (17.2, 19.6) | < 0.001 |
| Pulmonary artery systolic pressure, mmHg | 34.9 (29.8, 36.8) |  | | 29.8 (24.4, 38.0) | 0.04 |
| RV end-diastolic volume, ml | 45.6 (38.8, 52.5) |  | | 41.3 (35.8, 46.1) | 0.24 |
| RV ejection fraction, % | 34.2 (30.7, 38.5) |  | | 40.9 (36.6, 46.1) | < 0.001 |
| RVLS of free wall, % | -18.0 (-20.8, -13.5) |  | | -22.0 (-24.3, -18.7) | < 0.001 |
| RVLS of septal wall, % | -10.5 (-12.0, -8.9) |  | | -12.3 (-13.7, -10.9) | 0.006 |
| Operative data |  |  | |  |  |
| Concomitant MAZE procedures | 12 (38%) |  | | 2 (4%) | < 0.001 |
| Mechanical mitral prosthesis | 23 (72%) |  | | 39 (85%) | 0.25 |
| Cardiopulmonary bypass time, min | 97.0 (83.3, 120.0) |  | | 78.5 (66.8, 99.3) | 0.001 |
| MV lesion |  |  | |  |  |
| Stenosis | 26 (81%) |  | | 34 (74%) | 0.59 |
| Regurgitation | 3 (9%) |  | | 6 (13%) | 0.73 |
| Mixed | 3 (9%) |  | | 6 (13%) | 0.73 |

LV, left ventricle; MV, mitral valve; NYHA, New York Heart Association; RV, right ventricle; RVLS, right ventricular longitudinal strain; TAP, tricuspid annuloplasty; TR, tricuspid regurgitation; TV, tricuspid valve.

Data are expressed as median (interquartile range) or n (%).

**Additional Table 2.** Follow-up data in patients with less-than-moderate functional tricuspid regurgitation

|  | **One year follow-up data** | | | | |  | **Compared with preoperative data** | | | | |  | | **Two-way**  **ANOVA** | |
| --- | --- | --- | --- | --- | --- | --- | --- | --- | --- | --- | --- | --- | --- | --- | --- |
|  | **TAP Group**  **(n=32)** | **Non –TAP**  **(n=46)** | | ***P* value** | |  | | **TAP Group**  **(n=32)** | | **Non -TAP**  **(n=46)** |  | | **Interaction** | |  |
| NYHA functional class III-IV | 2 (6%) | | 1 (2%) | | 0.57 |  | | | < 0.001 | < 0.001 |  | | — | |  |
| Atrial fibrillation | 10 (31%) | | 11 (24%) | | 0.47 |  | | | 0.04 | 0.13 |  | | — | |  |
| Echocardiographic data |  | |  | |  |  | | |  |  |  | |  | |  |
| TR grade |  | |  | |  |  | | |  |  |  | |  | |  |
| None to trace | 22 (68%) | | 11 (24%) | | < 0.001 |  | | | < 0.001 | 0.03 |  | | — | |  |
| Mild | 6 (19%) | | 24 (52%) | | 0.003 |  | | | < 0.001 | 0.06 |  | | — | |  |
| Moderate | 4 (13%) | | 10 (22%) | | 0.30 |  | | | 0.13 | 0.002 |  | | — | |  |
| Severe | 0 | | 1 (2%) | | 1.00 |  | | | 1.00 | 1.00 |  | | — | |  |
| LV end-diastolic volume, ml | 92.5 (85.0, 107.3) | | 104.0 (90.5, 116.0) | | 0.21 |  | | | 0.26 | 0.54 |  | | 0.75 | |  |
| LV ejection fraction, % | 60.5 (58.0, 63.8) | | 61.0 (58.0, 62.3) | | 0.98 |  | | | 0.34 | 0.003 |  | | 0.21 | |  |
| Left atrial volume, ml | 77.1 (55.8, 107.5) | | 83.8 (57.8, 106.5) | | 0.52 |  | | | < 0.001 | < 0.001 |  | | 0.66 | |  |
| Right atrial volume, ml | 33.8 (28.1, 38.9) | | 38.6 (29.5, 44.6) | | 0.17 |  | | | 0.02 | 0.08 |  | | 0.004 | |  |
| Pulmonary artery systolic pressure, mmHg | 22.7 (18.8, 28.1) | | 24.7 (21.2, 28.1) | | 0.27 |  | | | < 0.001 | < 0.001 |  | | 0.06 | |  |
| RV end-diastolic volume, ml | 43.0 (32.6, 49.2) | | 48.0 (42.9, 57.4 ) | | 0.003 |  | | | 0.01 | < 0.001 |  | | < 0.001 | |  |
| RV ejection fraction, % | 39.0 (34.7, 42.7) | | 38.6 (34.3, 42.4) | | 0.86 |  | | | 0.001 | 0.001 |  | | < 0.001 | |  |
| RVLS of free wall, % | -18.4 (-19.9, -16.0) | | -19.2 (-20.7, -15.7) | | 0.41 |  | | | 0.74 | < 0.001 |  | | 0.001 | |  |
| RVLS of septal wall, % | -11.8 (-15.2, -10.5) | | -11.4 (-13.5, -8.9) | | 0.30 |  | | | 0.007 | 0.14 |  | | 0.002 | |  |

LV, left ventricle; NYHA, New York Heart Association; RV, right ventricle; RVLS, right ventricular longitudinal strain; TAP, tricuspid annuloplasty; TR, tricuspid regurgitation.

Data are expressed as median (interquartile range) or n (%).
